# Investigation of the Influence of Machining Parameters and Surface Roughness on the Wettability of the Al6082 Surfaces Produced with WEDM

**DOI:** 10.3390/ma17071689

**Published:** 2024-04-07

**Authors:** Dimitrios Skondras-Giousios, Panagiotis Karmiris-Obratański, Magdalena Jarosz, Angelos P. Markopoulos

**Affiliations:** 1 Laboratory of Manufacturing Technology, School of Mechanical Engineering, National Technical University of Athens, 15780 Athens, Greece; amark@mail.ntua.gr; 2 Advanced Manufacturing Laboratory, Department of Manufacturing Systems, Faculty of Mechanical Engineering and Robotics, AGH University of Krakow, 30-059 Krakow, Poland; karmiris@agh.edu.pl; 3 Department of Physical Chemistry & Electrochemistry, Faculty of Chemistry, Jagiellonian University, Gronostajowa 2, 30-387 Krakow, Poland; magdalenamjarosz@gmail.com

**Keywords:** WEDM, machining parameters, hydrophobicity, surface roughness, FEM, stochastic roughness parametric surface

## Abstract

Electrical Discharge Machining (EDM) is a non-conventional machining technique, capable of processing any kind of conductive material. Recently, it has been successfully utilized for producing hydrophobic characteristics in inherently hydrophilic metallic materials. In this work, Wire Electrical Discharge Machining (WEDM) was utilized for producing hydrophobic characteristics on the surface of the aluminum alloy 6082, and various parameters that can affect wettability were investigated. Adopting an orthogonal Taguchi approach, the effects of the process parameter values of peak current, pulse-on time, and gap voltage on the contact angles of the machined surfaces were investigated. After machining, all samples were observed to have obtained hydrophobic properties, reaching contact angles up to 132°. The peak current was identified as the most influential parameter regarding the contact angle, while the gap voltage was the less influential parameter. A contact angle variation of 30° was observed throughout different combinations of machining parameters. Each combination of the machining parameters resulted in a distinct surface morphology. The samples with moderate roughness values (3.4 μm > Sa > 5.7 μm) were found to be more hydrophobic than the samples with high or low values, where the contact angle was measured under 115°. In addition, the finite element modeling of the experimental setup, with parametric surfaces of uniform random and Perlin noise types of roughness, was implemented. Time dependent simulations coupling phase field and laminar flow for the modelingof the wetting of surfaces with different surface roughness characteristics showed that an increase in the Sa roughness and total wetted area can lead to an increase in the contact angle. The combination of experimental and computational results suggests that the complexity of the wettability outcomes of aluminum alloy surfaces processed with WEDM lies in the interplay between variations of the surface chemical composition, roughness, micro/nano morphology, and the surface capability of forming a composite air/water interface.

## 1. Introduction

The basis of surface wettability has been described long ago, and involves the effects of the triple-phase line of gas, liquid, and solid chemistry interaction [1], and the effects of the surface roughness of homogenous [2] or composite wetting [3]. However, only after the more recent discovery of bizarre anti-wetting mechanisms found in nature, such as the lotus leaf [4], water strider legs [5], or cicada wings [6], a great research interest in fabricating superhydrophobic surfaces has grown. Altering the surface wettability towards hydrophobicity is a highly desired surface modification that can be utilized in numerous applications that demand surface properties such as corrosion resistance [7], self-cleaning [8], anti-icing [9], or antibacterial properties [10]. Materials such as polymers that possess a low surface energy are naturally strong candidates in anti-wetting applications due to their inherent hydrophobicity [11]. On the other hand, materials with a low surface energy, such as metals, need surface modification in order to obtain hydrophobic attributes. Metallic materials are widely utilized in aerospace, automotive, and medical industries due to their high strength, durability, and biocompatibility. However, due to their poor anti-wetting properties, issues related to corrosion, icing, or bioadhesion are posing a challenge to overcome [12].

Various methods of inducing the hydrophobicity of metals have been employed, mainly based on chemical, mechanical, or thermal surface modification strategies. A straightforward way of modifying the wettability of an inherently hydrophilic material is by applying a hydrophobic coating. Such techniques include anodization [13], chemical etching [14], grafting [15], or hydrothermal synthesis [16]; these techniques aim to create a hydrophobic layer over the metallic surface. However, in some of these coating methods, issues arise, like environmentally hazardous substances or the durability of the coatings used. High precision surface machining approaches usually focus on creating hydrophobicity by tailoring the micro or sub micro roughness of the material surface. Diamond turning machining (DTM) was utilized in order to create grooves over micro-pillars on a brass-360 alloy surface, improving its hydrophobicity [17]. A hierarchical morphology on the surface of the AA 6061 alloy was created with vibration-assisted cutting, which resulted in greater hydrophobicity. When more primary manufacturing processes are utilized, it is more difficult to create a hydrophobic metallic surface. For that reason, they need to be combined with a chemical surface modification as an extra step. Along with stearic acid surface modification, a microgrooved aluminum surface, which was manufactured by common milling methods, gained hydrophobic properties [18]. A dual-scale anisotropic hydrophobic surface was realized by combining mechanical broaching and chemical oxidation [19]. The thermal modification of surfaces usually involves the creation of micro and sub micro features along with a slight chemical modification of the surface due to thermal effects. The most profound process is laser beam machining, although it is considered a high-cost manufacturing process. The creation of tailored micro-holes by efficiently manipulating nanosecond laser parameters induced a composite, Cassie–Baxter hydrophobicity regime on steel surfaces [20]. Ordered microstructures along with laser-induced periodic surface structures (LIPSS) were created using femtosecond laser processing, realizing hydrophobic steel structures [21]. Moreover, processes utilizing electron beam irradiation [22] or plasma arc [23] have been used along with other processes for increasing hydrophobicity.

A well-known non-conventional manufacturing process that has been only recently established as suitable for inducing hydrophobic properties in metallic surfaces is Electric Discharge Machining (EDM). A crater-like morphology is created all over the machined surface due to the formation of thousands of randomly distributed discharge channels during a pulse. Using Wire Electric Discharge Machining (WEDM), without any further chemical treatment, superhydrophobic surfaces were fabricated on AA 7075 samples, exhibiting a Cassie wetting state [24]. The dual-scale morphology surfaces can give a rise to hydrophobicity [25]. Such hierarchical surface textures can be fabricated by utilizing a textured electrode in sink EDM [26,27], by applying a coating from the electrode to the surface during discharging [28,29] or by controlling the wire cut path in order to create various geometrical features with WEDM [30,31,32]. Applications of hydrophobic surfaces created with EDM and WEDM include improved heat transfer surfaces [33,34] anti-bacterial surfaces [35], anti-fouling surfaces [36,37], corrosion resistance surfaces [38], and surfaces for anisotropic wetting [39].

Although such dual-scale morphologies can actually increase the hydrophobicity of the machined surface, the key factor of the wettability transition from the intrinsic hydrophilicity of metals towards hydrophobicity after EDM is the microroughness created. The machining parameters of the process control the derived microroughness, which in turn controls the wetting behavior of the surface. On a copper surface produced with WEDM and chemical surface modification for the enhancement of hydrophobicity, the increase in the pulse width and peak current led to a gradual decrease in the contact angle (CA) of specimens, while no correlation was found with the change in the pulse interval [40]. The current increase during the sink EDM of copper surfaces was reported to increase the CA, while surface roughness values (Ra and Rz) were found to increase and then decrease at higher currents (12 A) [36]. Using magnetic mixed EDM on Ni-Ti alloy surfaces for inducing hydrophobicity, it was found that with the increase in the pulse duration up to 90 μs, the roughness value Ra was also increased, while combining lower currents (1.5 A) and a moderate pulse duration (60 μs) was optimal for achieving greater hydrophobicity [41]. The effect that various machining parameters had on the CA and roughness of the AA 7075 surface that was fabricated using sink EDM was investigated [42]. It was found that the CA was decreased by using discharge duration values over 10 μs, while no correlation with roughness values was observed. On the contrary, a comprehensive study on the effect of a wide variety of roughness parameters with the CA on EDMed AA 6060 surfaces has shown a strong correlation for scales between 16 and 66 μm for curvature statistical parameters [43]. Hydrophobic SS 304 steel surfaces created with EDM exhibited higher hydrophobicity and became rougher when the current and tool feed increased [44]. A multi-scale titanium alloy surface that was fabricated with WEDM and high voltage-induced weak electric arc machining showed increasing and decreasing trends in the water CA as the low voltage current increased, peaking at 2 A [45]. Titanium alloy surfaces were reported to exhibit a higher water CA when machined with multi-pass WEDM, compared to conventional WEDM, with the difference being attributed to the more uniform surface finish that resulted from the multi-pass finishing process [46].

The modeling of the wetting phenomena on surfaces engineered with EDM is a challenging topic that, except from the inherent complicated nature of wettability on structured surfaces [47], involves the intricacy of the surface topography created by multiple discharges [48]. Dual-scale surfaces of Al 6063 [49] and EN-GJL-250 [50], fabricated with WEDM, have been modeled in terms of wettability, by employing a force balance numerical calculation model and simulated microroughness with a single or multi-pulse discharge model. However, models coupling microroughness and hydrophobicity were not developed. The modeling methods that have proven to be effective for investigating wettability in the presence of a rough surface morphology are molecular dynamics (MD) [51], lattice Boltzmann (LT) [52], and phase field (PF) methods [53].

Although the effect of process parameters on hydrophobicity has been occasionally studied, no work has systematically studied the combined effect of WEDM process parameters and microroughness parameters on the hydrophobicity of metallic surfaces. In this work, a study on the effect of process parameters and microroughness on the hydrophobicity of the aluminum alloy 6082 is presented. As well, the study tracks the transition of the material’s wettability from its initial hydrophilic state to a hydrophobic state. In addition, the results are coupled with computational modeling of the wetting of stochastic roughness surfaces, such as the ones created with WEDM, which constitutes a novelty in the field of the wetting of metallic surfaces fabricated with the WEDM method.

## 2. Materials and Methods

### 2.1. Material and WEDM System Parameters

The material that was used for all the experiments was the aluminum 6082 alloy, conditioned in a T6 temper. It fundamentally comprises aluminum, while magnesium (Mg) and silicon (Si) function as the pivotal alloying constituents. The conventional chemical makeup of AA6082 is delineated as fluctuating between 0.7% and 1.3% Mg and 0.4% to 1.0% Si. The detailed chemical composition of the alloy is presented in Table 1. Owing to its propitious strength-to-weight ratio, AA6082 is extensively utilized in structurally critical components across aerospace, automotive, and marine engineering domains. Aluminum alloys typically possess among the lowest surface energies among the most commonly used engineering metallic materials [54]. Thus, aluminum alloys can be considered excellent candidates among other metals for applications of wettability altering towards hydrophobicity.

The specimen surfaces for the needs of the analysis of the current study were fabricated by planar cutting on a precision WEDM system (Knuth Neospark B500, Hamburg, Germany). The WEDM system consists of 4 CNC-controlled axes, with X/Y feeds reaching up to 1000 mm/min and a maximum cutting capacity of 300 mm^2^/min. The generator can produce up to 10 A and the specified lower surface roughness Ra that can be realized is 0.8 μm. The dielectric fluid used in all cases considered was deionized water. The machining parameters of interest can be configured explicitly through the control panel. The wire material is molybdenum, and the wire diameter is 0.18 mm. A Taguchi factorial experimental design was employed for three factors and three levels, in order to examine the effect of the selected process parameters on wettability and surface morphology. According to the literature review presented in the introduction, the most influential parameters on wettability were the peak current (Ip) and pulse-on time (Ton). Thus, along with the gap voltage (SV), they were selected as the three factors for the experimental procedure. In order to obtain a diversity of roughness values, the upper limit of the parameters was about three times higher than the machines default values for the process of aluminum. The factorial design with the nine samples is presented in Table 2. Topographical and wettability parameters were the outputs of the experimental procedure. A flat, untextured sample was prepared via milling, serving the purpose of the “control” sample.

### 2.2. Surface Topography, Composition, and Wettability Evaluation

Surface topography was determined utilizing a TOPO 01P (IOS, Krakow, Poland) contact profilometer, endowed with an induction measuring head, and further characterized by a diamond tip, exhibiting a cone morphology with a 2 μm radius and a 90° apex angle. The investigative apparatus was structured with a confocal sensor, substantiated by a 130 μm range and an 8 nm vertical resolution. Employing a Gaussian filter, predicated upon Fourier transformation, specific cut-off lengths were delineated. This metric concomitantly dictated which components of the measured profile—or intrinsic surface components—would be assimilated as quantifiable surface roughness, whilst concurrently suppressing others. Topography analyses were conducted on an arbitrarily selected machined surface, utilizing a cut-off of 2.5 ls and a stipulated evaluation length of 1.25 mm. For the extraction of surface data, samples machined with the same conditions were measured 30 times. The surface topography was additionally captured using scanning electron microscopy (SEM) supplemented with energy dispersive X-ray spectroscopy (EDS) analyses for evaluating the elemental composition of the machined surfaces. Quanta 200 (FEI, Hillsboro, OR, USA) and Su 70 (Hitachi, Tokyo, Japan) models were utilized for obtaining the necessary data. The EDS analysis was utilized for the pinpointing and mapping of chemical elements of interest within designated micro-areas.

For the wettability evaluation of the surfaces, contact angle measurements were taken for all the samples. Before the CA measurements, the samples were cleaned in an ultrasound bath in ethanol, acetone, and water for 5 min each. All CA measurements were performed using an OCA25 goniometer with a TBA100 holding system (DataPhysics, Filderstad, Germany), equipped with an automatic dosing system and SCA20 1.0 software at an ambient temperature. The values of CA were measured using SCA20 software, where a contour analysis was performed. Deionized water was used for CA measurements of all the samples. Depending on the measurements, dosing volumes of 2 μL were applied with a constant dosing rate of 0.2-µL·s^−1^. Before photographing the deposited droplet, the waiting time was set to 30 s to prevent any dynamic effects. A total of 3–5 droplets on each sample were deposited and measured. Mean values were calculated for the contact angle value of each sample.

### 2.3. Finite Element Modeling for the Wetting of Rough Surfaces

In the present work, a three-dimensional time-dependent finite element approach, coupling the phase field method with a two-phase laminar flow is employed in the COMSOL 5.6 Multiphysics environment. The phase field method has demonstrated its versatility and accuracy in modeling the wetting of rough hydrophobic surfaces. Its ability to handle complex geometries, incorporate surface energy effects, and capture dynamic processes with a relatively low computational cost are a few of the key aspects that led to this choice, compared to similar formulations such as the Level Set or the Volume of Fluid methods. Moreover, two random surface generation approaches were utilized as an approximation for modeling the rough surfaces produced by a stochastic process such as WEDM.

**Figure 1:**
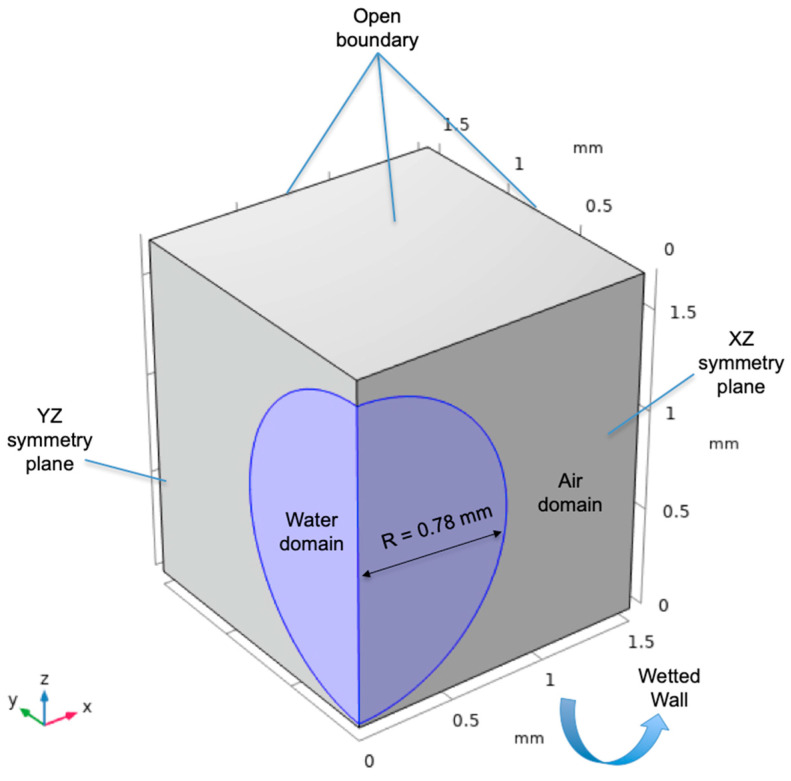
Geometrical setup of the model.

The general case that is going to be solved considers the impact of a water droplet at small velocities on a surface with different Sa values. The initial geometrical setup of the model is shown in Figure 1. The model dimensions are correlated with the values obtained from the 2 μL contact angle measurements of the previous sections. The model is built in a three-dimensional space consisting of water and air phases. The reason for selecting a three-dimensional space is that random roughness is affecting the measured contact angles in an unpredictable manner, as will be presented in the following sections. Open boundaries allow mass exchange and were selected for modeling an unconfined air space. The total volume has to be large enough in order to prevent the droplet interface to reach the open boundaries, as an unwanted mass exchange that causes numerical instability will occur. On the other hand, the volume must be sufficiently small for computational cost reduction. Considering these things, a cube with 1.5 mm sides is the space selected, with two symmetry planes for the modeling of a quarter of the droplet. Preliminary simulations confirmed that the space is sufficiently large to avoid contact between the droplet and the open boundaries after spreading.

The interface boundary tracking was modeled via the phase field method, by utilizing the Cahn–Hilliard equation through the minimization of the total free energy of the system:

(1) $$\frac{\partial \varphi }{\partial t}+u\nabla \varphi =\nabla \frac{\gamma \lambda }{\varepsilon^{2}}\nabla \psi$$

(2) $$\psi =-\nabla \varepsilon^{2}\nabla \varphi +\left(\varphi^{2}-1\right)\varphi$$

where *ψ* is a phase field help function. One of the most important phase field modeling parameters is the *ε* parameter that controls the interface thickness. The default value is half the maximum element size at the region of the interface. Values that are too large can create instabilities that cannot effectively capture the interface movement, while values that are too small can lead to numerical instabilities. The contact angle can be explicitly specified between the two fluids, dictating the wetting regime of the model. Concerning the modeling of multi-scale roughness, such as the roughness created with the WEDM process, the contact angle can be used as a boundary condition for the representation of the smaller scale wetting interactions between the participating phases that contribute to the contact line movement; this is similar to the way that the friction coefficient and adhesion can be used as boundary conditions in order to represent the smaller scale tribological interactions between two contacting solids in relative movement [55]. The contact angle boundary condition is applied on the wetted wall surface of the model, as shown in Figure 1. Moreover, a mesh refinement approach for the moving air/water interface was utilized for the adequately fine meshing of the triple line without increasing the overall computational cost of the simulations.

One of the main objectives of the modeling and simulations that were carried out in the present work was to investigate the effect of roughness on hydrophobicity. The scale of interest for the effect of hydrophobicity is the microscale, which is correlated with the Sa roughness measurements that were taken from the experimental section. Although hydrophobicity can also be affected by smaller morphological variations, in this section the effect of microscale roughness will be captured. The nature of the EDM process regarding surface morphology is considered highly stochastic [56] or even chaotic [57]. In that sense, for the modeling of roughness, cases utilizing uniform random and fractal noise generation approaches for the surface topography generation were selected.

**Figure 2:**
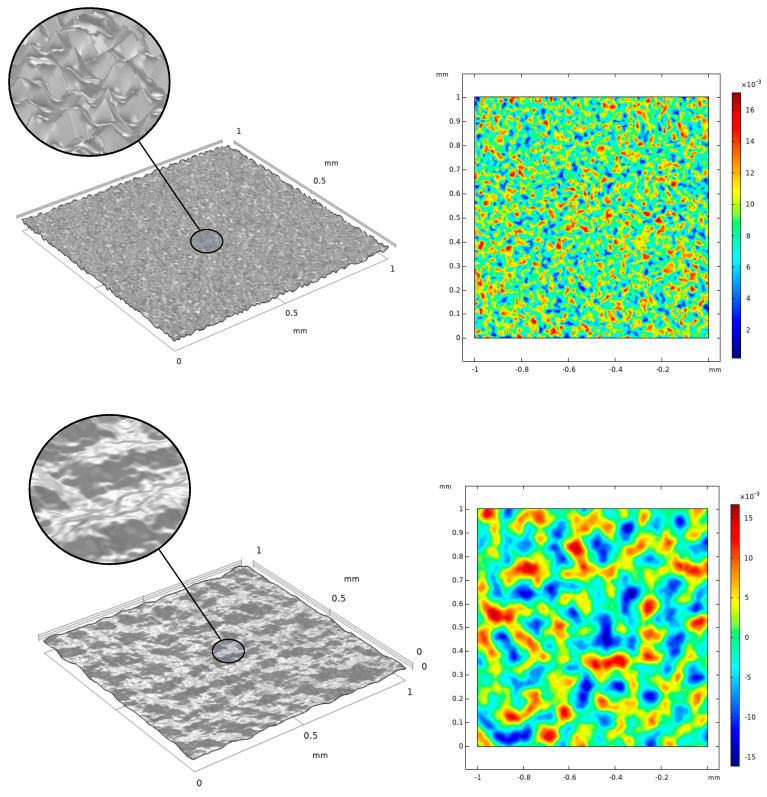
Uniform random (**up**) and Perlin noise (**down**) parametric surfaces with Sa = 4.5 μm.

For the generation of the uniform random surface, a data set of x, y points is created with the dimensions of the desired space A = [1,1] with a step of dx = dy = 0.01. Then, a uniform random function is multiplied with every point, representing the point height z. The next step involves the calculation of the arithmetic mean height of the surface Sa. By multiplying the height value of every point with a factor, the desired Sa can be found after the application of a simple iterative process for determining the value of the factor. In uniform random approaches, the surface points alter randomly between some minimum and maximum values. However, a “smoother” randomness can be introduced with the use of more sophisticated functions such as Perlin noise [58]. In the case of Perlin noise, coherence between adjacent values is introduced. Initially, the surface is separated into grid points. Pseudorandom gradient vectors are introduced for each grid point and distance vectors are created from each grid point towards a surface point inside the cell (2D) or cube (3D). Then, the four-dot product between the four gradients and distance vectors are calculated:

(3) $$z_{i}={gr}_{i}(x_{i}-x_{gri})$$

where ${gr}_{i}$ is the gradient vector, $x_{i}$ is the surface x-coordinate, and $x_{gri}$ is the x-coordinate of the grid point. The same procedure is followed for the y coordinates and for every surface point. In the last step, a smoothstep function $f_{sm}$ is used for the interpolation and generation of the Perlin noise [59]. The two types of parametric surfaces created in this work are shown in Figure 2.

## 3. Results and Discussion

In this section, the wettability transition of the initially hydrophilic 6082 aluminum alloy samples towards hydrophobicity will be discussed, considering the effect of surface energy alteration, different combinations of machining parameters, as well as the roughness parameter influence on the contact angle values of water droplets. To further investigate the effect of roughness on wettability, we conducted time-dependent simulations of water droplets—of the same size as those in the experimental cases—contacting surfaces with modeled random Sa roughness values similar to the Sa roughness values measured from selected machined samples.

### 3.1. Wettability Transition of the Hydrophilic 6082 Aluminum Alloy

**Figure 3:**
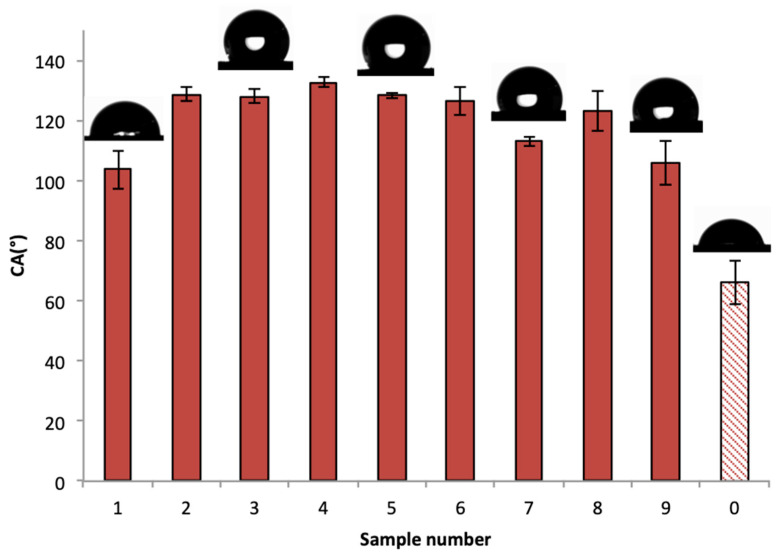
Contact angle measurements of all the samples for 2 μL droplet volume. The dotted column represents the sample before discharge machining.

To observe the wettability behavior after the machining of the samples, contact angle measurements were taken from all samples, as shown in Figure 3. The flat untextured sample 0 is hydrophilic, indicating the wetting nature of the aluminum alloy, which is hydrophilic. Contact angle measurements of the machined samples are all above 90°, showing a characteristic wetting transition from hydrophilicity to hydrophobicity. This wetting transition can be the result of the interplay between several mechanisms that were initiated due to the nature of the WEDM process.

The surface energy dictates the wetting state of a surface. Since the aluminum alloy utilized for this study is, like the vast majority of metals, hydrophilic, it is highly possible to consider that the alloy underwent a chemical transition after the machining process, which decreased its surface energy. In cases of processes involving extremely high temperatures, such as EDM or laser machining processes, the wettability transition is reported to gradually occur due to the improved ability of the oxidized surface to adsorb and react with carbon. In both processes, the increase in the amount of carbon in the metallic surfaces is considered the key factor that induces the wettability transition [29,60,61,62]. In the case of aluminum surfaces, the adsorption of carbon from carbon dioxide, carbon monoxide, and organic compounds found in the air has been proposed as the underlying mechanism of the hydrophobization of the surfaces [63]. More specifically, after EDM processes on aluminum alloy surfaces, it has been found that the C/Al atomic ratio has been increased, compared to untextured samples [32,42]. Typically, the wettability transition is completed after a period of a few days. After that period, the surface capacity for carbon adsorption is limited and no changes in wettability are observed.

**Figure 4:**
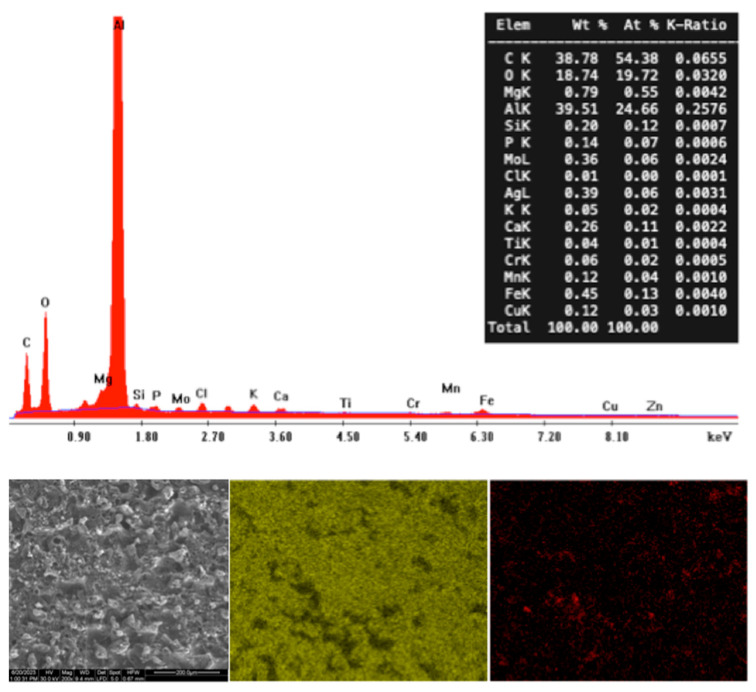
EDS spectra and mapping of Al (yellow) and C (red) elements for WEDM-processed aluminum alloy 6082 sample after the contact angle measurements.

Considering the above-mentioned observations, the contact angle of the samples was measured three weeks after being processed with WEDM. Following the contact angle measurements, an EDS analysis of the processed surface was performed in order to investigate possible chemical composition changes. A significant rise in the carbon content of the surface is observed from the elemental analysis, shown in Figure 4. The atomic percentage of carbon on the alloy surface increased to 54.38% after a period of approximately one month after being processed with WEDM. This is a strong indication that the machined surfaces underwent a wettability transition. The typical ultrasonic cleaning procedure before the contact angle measurements may have also contributed to the increased carbon content. The cleaning process includes cleaning steps in acetone and ethanol solutions. Acetone and ethanol are organic compounds; thus, a part of the carbon increase on the alloy surface might also be attributed to organic adsorption. It is important to note that the preliminary contact angle measurements that were performed on the processed samples after a week showed superhydrophilic results. This observation agrees with some of the above-mentioned studies that reported an initial intense hydrophilic wetting state of the aluminum surfaces. In all the cases considered in the present study, the delay in the wettability transition is more likely attributed to the fact that deionized water was used as a dielectric liquid. In cases where the dielectric fluid is oil, which is typically carbon-based, the wettability transition can instantaneously occur, due to the increased carbon content on the machined surfaces [42]. The surface energy of the machined surfaces could also be affected by changes in the intermolecular forces of the surface. Although the white layer can exhibit phase changes that could possibly affect the surface intermolecular forces, the fact that the wetting transition did not occur instantaneously after the discharge machining indicates that other factors subsequently affected the surface wettability. Accordingly, the detection of the electrode material in the machined surface cannot be considered to drastically change the surface energy. Molybdenum is not considered to be hydrophobic. In general, the delay in the wettability transition is a sign that any instantaneous surface composition changes due to discharge machining did not significantly affect the surface wettability.

Without such a transition, the effect of roughness and texturing on a hydrophilic surface would have the effect of further increasing the wettability. For inherently hydrophilic surfaces such as metallic surfaces, a wettability transition is a crucial phenomenon that allows topographical effects to have a beneficial effect on hydrophobicity. The surface after the discharge machining process has a quite unique morphology, which is a qualitative characteristic of the process. The size and intensity of the morphological features involved are unique for each combination of materials, which exhibit a different response through varying machining process parameter intensities that utterly affect the plasma channel intensity and size.

### 3.2. Process Parameters and Surface Topography Effect on Wettability

The process parameters can drastically affect the final surface topology, which in turn affects the wettability outcome in each case. The results of the surface roughness parameters and contact angle values for the nine different combinations of the WEDM process are depicted in Table 3, according to the L9 Taguchi design of the experiments. The different process parameters had an effect on most of the surface roughness parameters as well as the contact angle values. Regarding the higher order areal roughness parameters of skewness and kurtosis, Ssk and Sku, the effect of different process parameters did not result in any significant variation. Thus, it can be considered that the wettability of the surfaces machined in this work is not affected by alterations of higher order surface roughness parameters.

**Figure 5:**
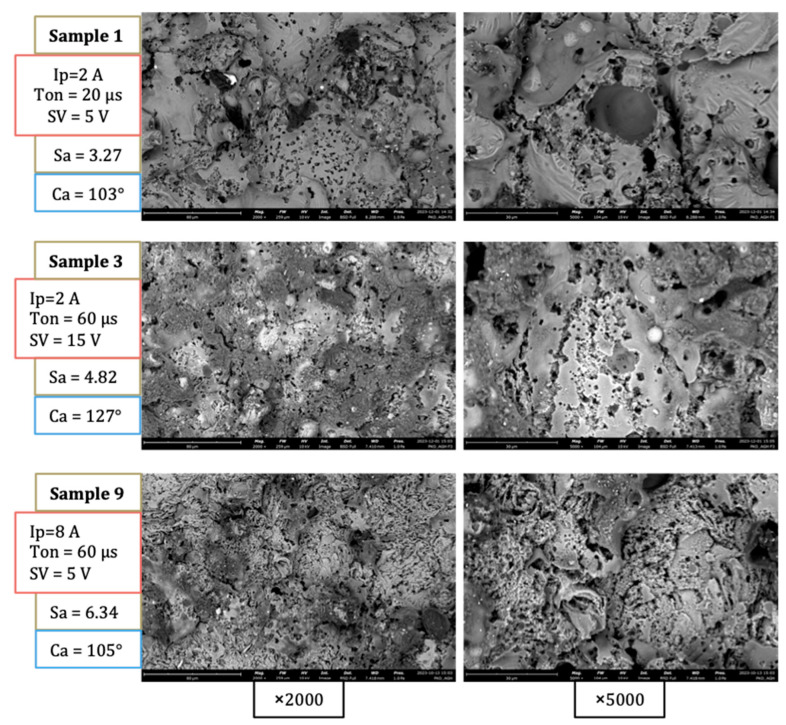
SEM images of samples 1, 3 and 9.

The effect of the different combinations of the process parameters can be qualitatively observed from the SEM images of the surfaces of samples 1, 3, and 9, presented in Figure 5. The surface of sample 1 is the result of milder machining parameters than the other two samples. This surface has the largest smooth recast layer area compared to the other two sample surfaces, which are filled with porous areas.

On the contrary, sample 9 was produced by the most intense machining parameters among all the samples. The majority of the surface is covered by a rough morphology. The rough morphology of the surface of sample 9 is below the scale of craters and can be attributed to the superposition of overlapping resolidified layers. On the surface of sample 3, an intermediate morphological regime can be observed. Smooth and rough regions can be observed, while porosity is evident, as in sample 1. Combined with the wettability transition that the surface underwent, the air entrapment mechanism can further increase the hydrophobic outcome of a surface, creating areas where the Cassie–Baxter interface is present. From this perspective, the discharge process formation of the micro/nano morphology can be considered a favorable factor for reducing the machined surface wettability [43,64,65].

**Figure 6:**
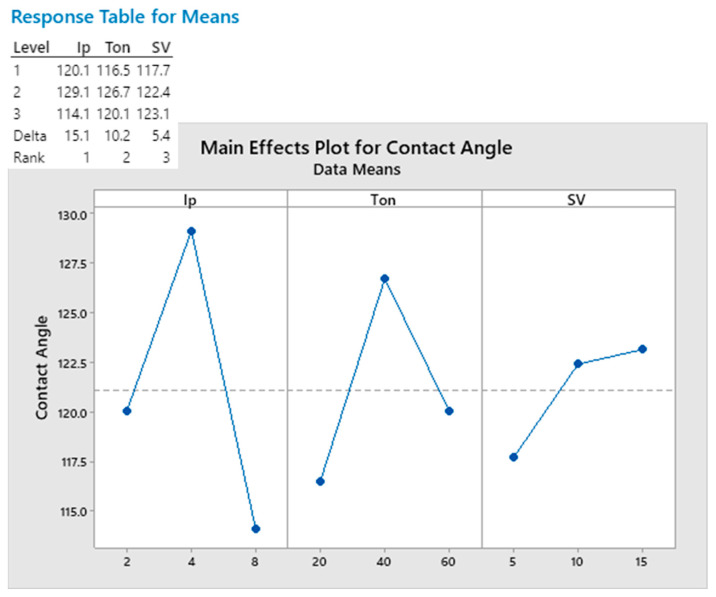
Main effect plot and response table of process parameters on contact angle.

The influence of the process parameters on wettability can be seen in the main effect plots of the Ip, Ton, and SV parameters on the contact angle, which are presented in Figure 6. The variation in process parameters does affect the surface wettability of the samples. The SV parameter has the smallest influence on the wettability of the surface. However, the Ip and Ton parameters have a more significant effect, and the same trend regarding the variation in contact angle values with changes in those parameters is observed. Moderate values between the limits of the utilized process parameter values result in higher contact angle values. The peak current value is the most significant process parameter for controlling wettability compared to the other two process parameters. The Delta value of the response table covers more than 50% of the contact angle maximum deviation, which was observed from the experimentally obtained values. The peak current and pulse duration have been shown to affect the contact angle of surfaces processed with WEDM or EDM with varying trends. There is no study that specifically explores the effect of process parameters on the wettability of aluminum alloys. Studies on the relation between process parameters and the wettability of titanium alloys have observed a contact angle rise with an increase in the pulse duration up to 60 μs [41,66].

The control of the process parameters during the WEDM process can result in different intensities of hydrophobicity of the surfaces. The selection of the best combination of the process parameters can lead to an increase of up to 30° in the water contact angle, according to the obtained results. However, to further identify other factors that can affect wettability, the relation between roughness and contact angle was investigated.

**Figure 7:**
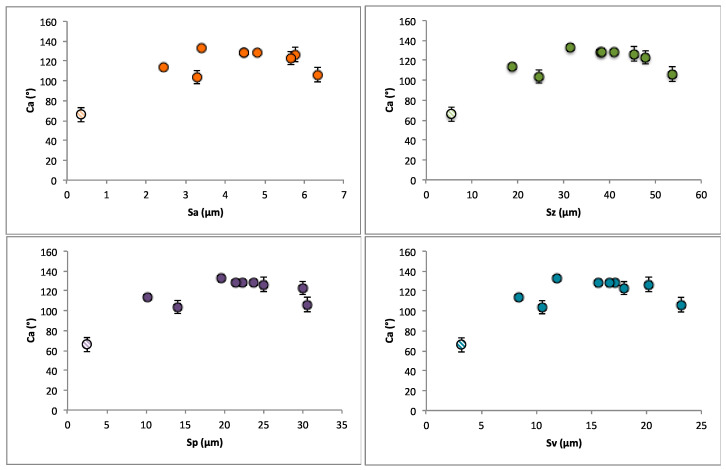
Plots of the variation of the contact angles of 9 samples and the untextured surface (lighter colored points) with surface roughness parameters (Sa, Sz, Sv, Sp). Dotted points represent the sample before discharge machining.

**Figure 8:**
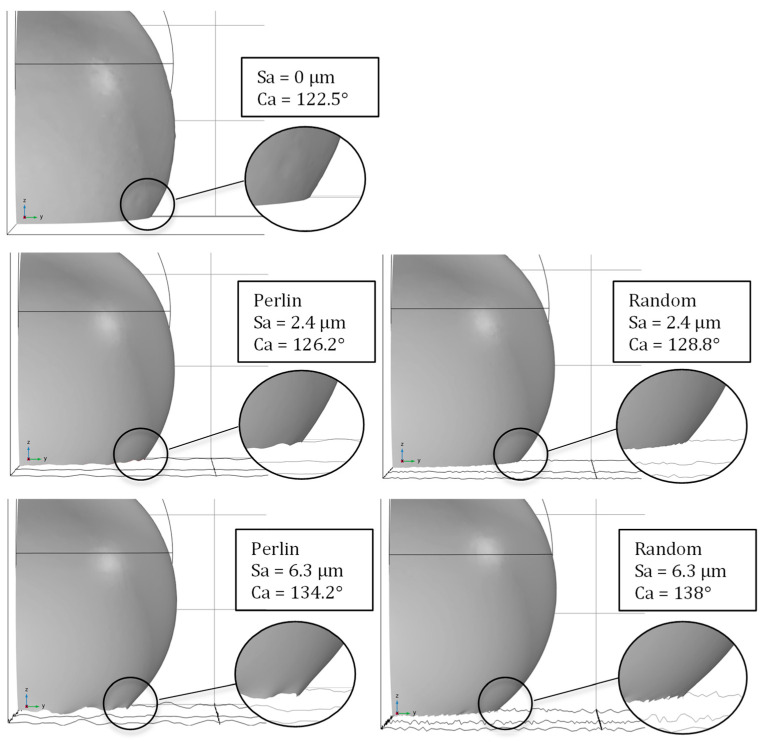
Snapshots of the simulations showing measured contact angle at 4.5 ms for different parametric surfaces and Sa values.

**Figure 9:**
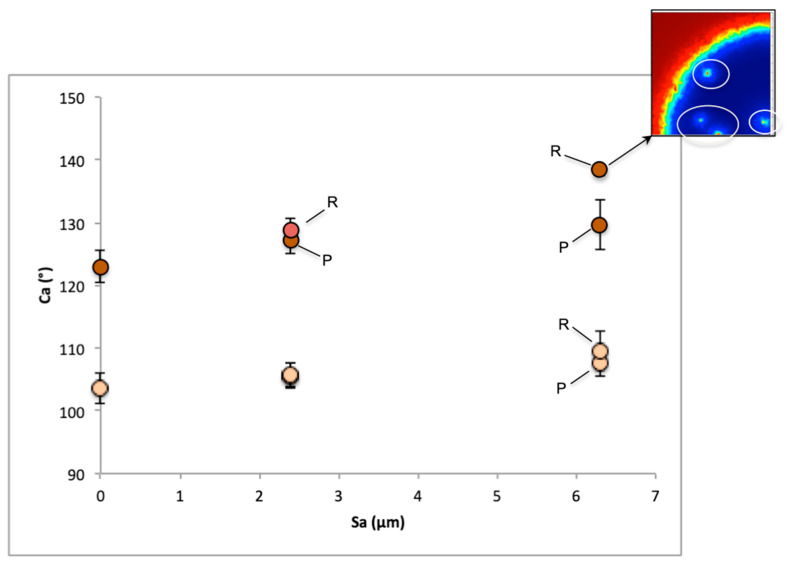
Contact angle values with increasing Sa for two different intrinsic hydrophobic wettabilities (lighter and darker points). The volume fraction plot (top right) represents the phase of air (red) and the phase of water (blue) on the wetted wall. Circled areas represent trapped air inside the water domain.

Surface roughness can play a key role in surface wettability. The relationship between Sa, Sz, Sp, and Sv and the measured contact angles of the surfaces can be seen in Figure 4, Figure 5, Figure 6, Figure 7, Figure 8 and Figure 9. Samples with moderate values of surface roughness parameters exhibit higher contact angle values for all parameters. Surface roughness values that are too high or too low display lower contact angles, considering the mean variation from the mean line of the peak and valley surface measurements. The same trends have been observed in surface textures with an electric discharge. Given that the theoretical maximum of water on an ideally smooth surface is at 120° [67], the fact that the roughness leads to the forming of a homogeneous or composite wetting state can be safely considered as a factor of the increase.

On the adjacent margins of the roughness values produced, the contact angles have shown an initial increase followed by a decrease to some extent, without losing their hydrophobic character [36,41,68]. The margins of roughness that were reported were different in the case of the EDM of copper [36] and the Ni-Ti alloy [41]; in the case of 5083 aluminum alloy, they were almost identical, up to 6μm [68]. In other experimental works concerning EDM processes, the contact angle was found to increase [43] or slightly increase [62] with the increase in roughness. In most of those works, the hypothesis was that the surface morphology is responsible for creating more air pockets that increase in volume with the increase in roughness, until a critical point is reached, after which the volume of the cavities is no longer in a position to retain air. After that critical point is reached, a transition from a Cassie–Baxter composite interface to a Wenzel regime occurs, leading to a small contact angle decrease. However, the experimental observation of the air-trapping mechanism is difficult to realize and is not yet employed in the relevant literature. In addition, the carbon adsorption of the machined specimens can be different even when machined under the same conditions [69]. Therefore, contact angle predictions according to Wenzel or Cassie regimes are not considered to be a helpful tool, as the intrinsic surface energy of the surface cannot be explicitly determined. The reason is that the generated surfaces incorporate a combined change in surface energy and roughness. Moreover, even if multiple cuts are used to produce a smooth surface for the purpose of a surface energy reference, composition changes may still occur between the number of passes [70].

### 3.3. Simulation Results of Droplet Wetting on Rough Surfaces Produced by WEDM

In the previous section, the complicated wetting outcome that involves contributions from surface energy changes, the ability of micromorphology to trap air and the roughness amplitude increase, were pinpointed. The aim of the model was to present the variation in the contact angle wettability metric with changes in the surface roughness of wetted walls with distinct surface energies. Another aim was to investigate the effect of roughness on wettability, isolated from the effects of air-entrapment inside nanoscale morphologies or surface energy inhomogeneity. Twelve final time-dependent cases of droplets wetting the surface from a fixed small height of 0.02 mm with different combinations of Sa and intrinsic contact angle values were considered, as presented in Table 4, for the two-type parametric surfaces. The highest and lowest Sa values obtained from the experiments were selected as the two different surface roughness amplitudes, while high and low contact angles represented different surface energies, and thus different wettability intensities. The lowest value of surface energy, which was modeled with a higher contact angle value on the wetted wall boundary, represents a theoretically extremely hydrophobic flat surface or a surface forming a stable composite air/water interface. Considering the dimensions of the model, the Weber number is <<1; therefore, surface tension forces dominate over gravity in the simulations that took place. The contact angle calculation was performed in selected time steps by explicitly calculating the angle of the component of the z normal direction of the interface with the XY plane increased by 90° at every point of the triple line of the air, water, and surface. Then, the mean value of all points was calculated to extract information about the whole triple line average contact angle.

The contact angle values of the points of the triple line did vary in value; thus, the selection of a 3-D model is justified. In a 2-D case, only one point would be considered, instead of the thousands of points that were considered in the 3-D case. Thus, the contact angle that results from a 2-D model could be highly unreliable.

Preliminary simulations were performed for the fine-tuning of the model, examining the importance of various parameters on the accuracy of the solution and relative computational cost. Simulations with different slip length parameters, namely 0–0.001 mm, showed a deviation of ±0.26° in contact angle values, which is considered negligible. The small Re number can explain the low importance of the slip length in the current simulations. The parameter of controlling interface thickness epf values was set sufficiently low, near the length scale of roughness, at 5 μm, which is much lower than the default (30 μm). Smaller values of 3 μm and 1 μm did not show any difference in contact angle measurements, showing a deviation of ±0.07°. However, the computational time was increased when employing smaller epf values.

In Figure 8, snapshots of the simulation at 4.5 ms are illustrated. Compared to the flat surface, all parametric surfaces with roughness exhibited higher contact angles. In surfaces with higher Sa values, the measured contact angle is increased. The cases with increased contact angles are observed to hinder the droplet movement. The triple line between the air, water, and surface spreads more freely with decreasing Sa values, while at higher roughness values, it is more restricted. Between the two types of parametric surfaces at the same Sa values, differences in contact angles are also observed. The uniform random type of parametric surface exhibits higher contact angles than the Perlin parametric surface. This difference can be attributed to the difference in spatial distribution along the XY plane. The uniform random surface possesses a higher spatial frequency of roughness distribution compared to the Perlin surface, as seen from the surface plots of Figure 2. This difference results in a more intense overall surface roughness result. Thus, the triple line undergoes more height variations over the same spreading length. This results in less spreading for a higher spatial frequency roughness, as observed in the simulations for the uniform random surface. At hydrophobic wetting states, the increased roughness makes it less energetically profitable for the droplet to spread, thus increasing its contact angle.

The evolution of contact angles for all cases is presented in Figure 9. The contact angle values represent the mean contact angle values of the output times of the simulation of each case, for the two different surface energy surfaces. An increasing trend in the contact angle was observed with the increase in Sa in both surfaces with different wettabilities. However, the trend for the surface with higher energy is more pronounced as the Sa value increases, compared to the one with the lower surface energy. The increased surface energy can lead to more intense droplet retraction and minimize spreading [71]. When combined with a rougher surface profile, then both account for an even higher droplet retraction and higher apparent contact angle [72]. As already noticed, the two different roughness distributions differ in the spatial frequency distribution. This difference is reflected in the contact angle values. In the cases of surfaces with smaller Sa values, the difference in the contact angle is quite low. More pronounced differences occur in the cases of both higher Sa values and surface energy. Regarding hydrophobic surfaces, the effect that increased roughness intensity has on the contact angle can be seen as similar to the friction mechanism [73]. Higher roughness will qualitatively result in lower wettability. Moreover, air entrapment was observed during the simulation of the surface with a higher surface energy, Sa value, and spatial frequency, as shown in the volume fraction plot over the wetted wall in Figure 9. This could also be a reason for the higher observed mean contact angle, although the area of the air fraction is relatively low to support this hypothesis, considering that air entrapment should be accounted for only in the triple phase line. In addition, this is an indicator that a combination of high surface energy, along with intense height and spatial distribution is needed to initiate the composite Cassie–Baxter regime. It can be suggested that if the composite interface was occurring on the surface of the experimentally machined samples of the previous section, it was most probably created over micro or nano-morphological variations such as cavities of the surfaces. Thus, craters with dimensions of decades of microns and small aspect ratios are highly unlikely to induce a composite surface.

To further examine the effect of roughness, the dimensionless factor of roughness *rf* is introduced and calculated for the meshed surfaces of the wetted walls of the models over surface areas of 1 mm^2^. The factor is the ratio of the actual surface area $A_{r}$ of the wetted surfaces to their projected surface area $A_{p}$:

(4) $$r_{f}=\frac{A_{r}}{A_{p}}$$

The roughness factor is used for the representation of the dimensionless actual surface area that is wetted for each simulation case. An increase in the roughness factor implies an increase in the roughness amplitude. Mesh refinement did not alter the mesh area on the wetted wall, as it was a priori meshed finely. Thus, *rf* is considered to be constant during each simulation. In order to examine the mean contact angle values of cases with roughness between the two Sa values, two extra cases were simulated for the higher surface energy surface, and Sa = 4.5 μm for the two types of parametric surfaces. This value was chosen in order to represent a moderate experimentally observed value between the maximum and the minimum previously selected values. The calculated values for the roughness factor for the cases selected are presented in Table 5.

**Figure 10:**
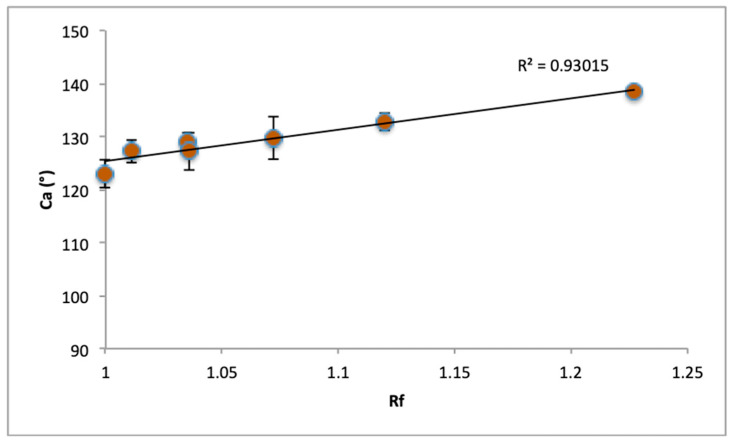
Plot of contact angle values with roughness factor *rf*.

The mean contact angle values for the cases shown in Table 5 are plotted against the roughness factor in Figure 10. A good correlation of contact angle values with the roughness factor is observed. As expected, the difference in the spatial frequencies of the different types of parametric surfaces is also significant to the contribution of the contact angle. The contact angle is highly dependent on the wetted area. An increase in the wetted area will lead to an increase in the contact angle value. This dependence can partially explain the difference in contact angles of the experimental roughness measurements. However, the involvement of the overall morphology on wettability is not so straightforward and cannot be accurately explained.

Considering the results from this section, a better insight can be given for the interpretation of the experimental results. The simulations suggest that the effect of increasing roughness could have a positive effect on amplifying hydrophobicity, especially as the surface energy decreases. However, the experimental contact angle values were not following that trend. Therefore, other phenomena are indeed likely to have collective implications for the overall wettability result. The surface energy deviation of the samples is one possibility that could overcome the effect of roughness, as seen from the simulation results. A higher surface energy can lead to the minimization of the intensity of the roughness effect on hydrophobicity, as seen in Figure 9. Thus, samples with higher roughness values but higher surface energies could induce lower contact angles than the opposite combinations. A more straightforward interpretation of the wettability result is the formation of composite air/water interface areas inside the micro/nano cavities, which can increase the apparent contact angle. Since machining parameters can influence the porosity in that scale, as analyzed in Section 3.2, the case of increased surface roughness in the microscale combined with fewer cavity areas can result in lower contact angles. Consequently, although the increase in roughness would have a positive effect on hydrophobicity, the minimization of surface cavities that can support air entrapment will lead to the mitigation of the contact angle increase. The effect of such nano-scale cavities is not considered in the simulations, due to their very fine scale, which is not captured in the surface modeling. Finally, the consideration of the total wetted area should be considered, as indicated from the simulation results. In a crater-like morphology such as that of the surface derived from the WEDM process, the total surface area can be altered non-monotonically with the machining parameters, regardless of the roughness values, when different machining conditions are applied. As seen from the simulation of the wetting of two different types of parametric surfaces, the total wetted area can be different even for surfaces with the same Sa value, leading to different wettability results.

## 4. Conclusions

In this work, an experimental investigation was conducted on the effect of different machining parameters on the wettability of aluminum alloy 6082 surfaces, considering the wettability transition, morphology, and roughness parameters of the machined samples. The effect of roughness and surface area on wettability was further investigated by employing a finite element analysis with coupled phase field and laminar flow modeling for selected surfaces of experimentally derived cases, with modeled parametric microroughness. The most important results derived are as follows:

- The WEDM process can effectively induce a wettability transition towards hydrophobicity for all machined samples, compared to the initially hydrophilic state of the aluminum alloy surface, altering the surface chemistry by the addition of carbon, as the EDS results showed.
- The contact angles of the machined surfaces can be qualitatively controlled via the Ton, Ip and SV machining parameters. The results showed that the most influential factor regarding wettability was the Ip parameter. Moderate values of the machining parameters within the experimental values tested exhibited lower wettability on the metallic surfaces. The selection of the optimal combination can lead to a rise of about 30° in contact angle values.
- Moderate values of the main roughness parameters tested were found on the most hydrophobic samples, according to the experimental results. This can be an indication that certain roughness parameters could be used for an initial estimate for the extent of the wettability reduction of the surfaces.
- Computational results showed that increased roughness and total contact area contribute to the increase in the contact angle values of the surfaces. The inclusion of a stochastic roughness distribution instead of directly using a flat surface with lower wettability is essential for accurately capturing the wettability aspects of surfaces produced using WEDM.
- The wettability outcome of the machined surfaces is a complex result of the interplay between the final chemistry composition, surface roughness, and morphology of the machined samples. The formation of a composite air/water interface is crucial for the further increase in hydrophobicity and results from the micro/nano morphology of the machined surface.

The combination of experimental and simulation results indicates that although the wettability of the aluminum alloy 6082 can be roughly controlled from the machining parameters of the EDM process, factors related to the surface structure such as roughness, morphology, and total wetted area should be considered for the evaluation of the surface wettability. Chemical composition variations in the surfaces produced by different machining parameters in the WEDM process should also be taken into account.

## Figures and Tables

**Table 1:** Chemical composition of AA 6082.

Al	Mg	Mn	Fe	Si	Cu	Zn	Cr	Ti
Bal	0.9	0.7	0.5	1.0	0.1	0.1	0.2	0.11

**Table 2:** L9 orthogonal array for WEDM process parameters.

No	Ip (A)	Ton (μs)	Sv (V)
1	2	20	5
2	2	40	10
3	2	60	15
4	4	20	10
5	4	40	15
6	4	60	5
7	8	20	15
8	8	40	5
9	8	60	10

**Table 3:** Surface topography parameters and contact angle of the machined samples.

Sample	Sa [μm]	Sz [μm]	Sp [μm]	Sv [μm]	Sq [μm]	Ssk	Sku	Ca [°]
1	3.277	24.563	14.063	10.5	4.08	1.557	2.808	103.61
2	4.483	37.93	22.325	15.605	5.586	1.575	2.917	128.63
3	4.822	40.974	23.777	17.196	5.981	1.576	2.967	127.95
4	3.404	31.486	19.62	11.866	4.285	1.609	3.08	132.72
5	4.466	38.153	21.528	16.625	5.59	1.606	3.075	128.33
6	5.766	45.28	25.022	20.258	7.144	1.556	2.822	126.34
7	2.447	18.602	10.174	8.428	3.069	1.627	3.11	113.15
8	5.672	47.913	29.963	17.95	7.1	1.606	3.07	123.16
9	6.341	53.727	30.61	23.118	7.928	1.615	3.124	105.87

**Table 4:** Parameter combinations for simulation cases.

Cases	Sa [μm]	Ca [°]
1	0	103
2	2.4	103
3	6.3	103
4	0	123
5	2.4	123
6	6.3	123

**Table 5:** Roughness factor values for the meshed areas of wetted walls.

**Sa [μm]**	0	2.4		4.5		6.3	
**Type**	Flat	Perlin	Random	Perlin	Random	Perlin	Random
***rf***	1	1.011	1.035	1.036	1.12	1.072	1.227

## Data Availability

Data are contained within the article.
